# Electroporation outcomes in human Jurkat cells are affected by exposure to simulated microgravity

**DOI:** 10.21203/rs.3.rs-10896773/v1

**Published:** 2026-09-15

**Authors:** Matthew T. Conway, Anna V. Sedelnikova, Edward A. Sander, Kristan S. Worthington, Zachary A. Steelman

**Affiliations:** 1 University of Iowa, Roy J. Carver Department of Biomedical Engineering, Iowa City, IA, USA; 2 Science Applications International Corporation (SAIC), JBSA Fort Sam Houston, TX, 78234, USA; 3 Texas A&M University, Department of Biomedical Engineering, College Station, TX, 77843, USA

## Abstract

Electroporation (EP) is commonly used to permeabilize cell membranes to deliver nucleic acids and other biomolecules. EP-based methodologies have the potential to support in-space biomanufacturing; however, it is currently unclear whether cellular adaptations to microgravity influence EP outcomes. To investigate this, human T-lymphocyte (Jurkat) cells were exposed to simulated microgravity (SμG) in a rotating wall vessel (RWV) bioreactor prior to EP to deliver exogenous fluorescent molecules or plasmid DNA. We observed that two hours of SμG exposure led to a 20% decrease in fluorophore uptake and a 31% decrease in transfection efficiency compared to standard gravity controls for several voltages. To explore underlying mechanisms, the effects of SμG on actin organization were assessed using a phalloidin stain, which revealed increased cell area and cortical actin thickness following SμG. Actin targeting drugs jasplakinolide and latrunculin B were used to promote or destabilize F-actin, respectively, and modulated cell area and cortical actin thickness. Promoting F-actin also led to decreased transfection efficiency, mimicking the effect of SμG, while destabilizing F-actin during SμG showed a small increase in transfection efficiency, slightly reversing the impact of SμG. Our results demonstrate that adaptations to microgravity exposure can influence transfection efficiency via cytoskeletal reorganization.

## Introduction

In-space biomanufacturing (ISB) is an emerging field in space biology that utilizes living systems in combination with the reduced gravity environment of space to produce biomolecules [1, 2]. Microgravity conditions are known to impact the biophysical behavior of molecules in ways that may be advantageous for manufacturing, resulting (for example) in enhanced protein crystal structure and purity [3]. Logistically, ISB may also eventually be leveraged to supply neighboring orbiting space stations with specialized polymers, food, and medicines, which could alleviate supply-chain bottlenecks that limit the availability of resources in space [4-7]. However, ISB currently requires significant resource support from Earth to establish specific and reliable manufacturing processes during spaceflight [8]. Given the extreme costs associated with launching payloads [9], there is demand for multi-use, mission-adaptable, and in situ biomanufacturing tools in the space domain.

Electroporation (EP) is a potentially useful biotechnology for ISB and for enhancing space operations in general. During EP, new plasmid DNA is delivered to a cell via temporary permeabilization of the cell membrane using electric pulses. EP could help to establish tunable cellular engineering processes for ISB via the controlled delivery of DNA encoding proteins of interest, to be delivered to cells when mission requirements arise. This concept could substantially reduce the number of ISB pipelines needed to support life, since delivering an array of plasmid DNA samples is much more efficient than launching multiple distinct strains or bioreactors. EP is also of particular interest because it could serve as an efficient alternative to viral-based transfections [10], which could pose health risks to crew members during spaceflight [11]. Importantly, EP can also be used for disinfection [12, 13], purification, and biomolecule extraction [14], which are all important concepts in ISB and in supporting sustainable, long-term spaceflight.

While EP is a desirable tool to support space biosciences, there is a gap in knowledge regarding the relationship between cellular adaptations to microgravity and EP outcomes such as transfection efficiency and cell viability. EP outcomes are dependent on the overall health of the cell line under normal conditions [15], but in microgravity, cellular adaptations to changes in the mechanical environment can influence how cells respond to EP [16]. For example, in yeast it has been shown that exposure to simulated microgravity (SμG) for 24 hours led to significantly increased viability following 20 kV/cm millisecond pulses [17]. In our previous work, we demonstrated a decrease in uptake of fluorescent molecules following EP of human T-lymphocytes (Jurkats) that were exposed to SμG for two hours [18]. Preliminary evidence further suggests that microgravity exposure can alter transfection efficiency using other approaches. For example, an experiment performed aboard the International Space Station (ISS) used lipofectamine to deliver plasmid DNA to cells and showed that transfection efficiency was significantly reduced compared to ground controls [19]. Complicating matters, HepG2 and A549 cells exposed to SμG for 24 hours showed enhanced transfection efficiency through polymer mediated delivery of siRNA [20]. Given these studies, it is not comprehensively understood whether microgravity induces an increase or decrease in transfection efficiency, the extent to which transfection efficiency depends on cell type, and by which mechanism an altered mechanical environment is translated into a distinct transfection outcome.

Cytoskeletal remodeling in response to microgravity is a potential mechanism behind changes in transfection efficiency. In many cell types, microgravity exposure causes the cytoskeleton to reorganize in response to changes in the mechanical environment [21] leading to changes to cell division, adhesion, motility, and stiffness [22-25]. Cytoskeletal adaptations to microgravity can include changes in the organization and expression of F-actin, microtubules, and vimentin filaments. These adaptations are dependent on cell type and time spent in microgravity, which ranges from hours to days in published studies [26, 27]. Furthermore, manipulation of the cytoskeleton using small molecule inhibitors can drastically reduce uptake of exogenous molecules [28] and transfection efficiency [29, 30]. Elucidating the extent to which the cytoskeleton reorganizes during microgravity exposure and correlating these changes to uptake and transfection efficiency is therefore critical for developing EP as a tool for ISB.

In this work, we investigated the impact of SμG exposure in a rotating wall vessel bioreactor (RWV) on EP endpoints such as fluorophore uptake, cell viability, and transfection efficiency. A human T-lymphocyte cell line (Jurkat) was chosen for this study as Jurkat cells can be cultured in suspension and are known to be influenced by mechanical stimulation [31] and microgravity conditions [32]. To analyze the effects of SμG on Jurkat cells, we electroporated them in the presence of fluorescent dye or plasmid DNA to determine the fraction of cells stained with the dye or that were successfully transfected. Cells were exposed to SμG for 2 or 24 hours to investigate how EP endpoints such as transfection efficiency and viability change in response to different durations of SμG exposure. Finally, we investigated changes in cytoskeleton reorganization following SμG exposure and used F-actin targeting drugs to model how these effects can alter transfection efficiency in both standard and SμG conditions. The goals of this study are to determine the extent to which EP-mediated transfection is altered given exposure to SμG and to characterize the role of F-actin in the reorganization of the cytoskeleton in this scenario. Our results suggest that exposure to SμG reduced exogenous molecule uptake and transfection efficiency, and that actin reorganization is implicated in this behavior.

## Results

### Simulated Microgravity Exposure Reduces EP-Mediated Uptake of Fluorescent Molecules

**Figure 1.**
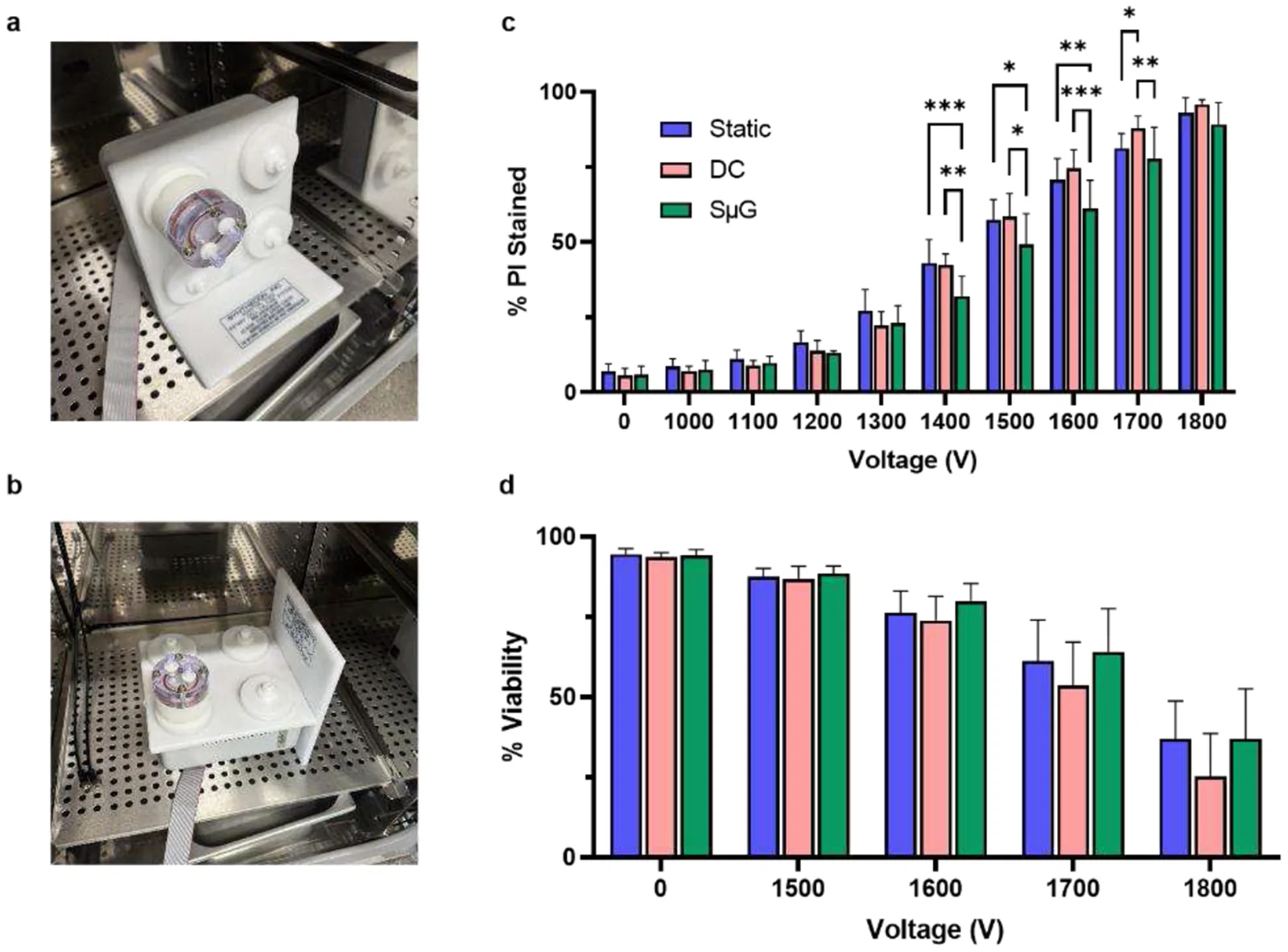
Jurkat cells exposed to SμG for two hours show decreased uptake of PI but unchanged viability following EP compared to standard gravity controls. a) RWV bioreactor with a volume of 4mL in the upright position for SμG exposure and b) RWV tilted on its side for DC exposure group to account for dynamic fluid motion within the rotating vessel while in standard gravity. c) EP-induced uptake of propidium iodide across a voltage ramp ranging from 0 to 1800 V, showing the percentage of cells staining positive for PI 30 minutes following EP. For each voltage, cells were pulsed three times with a 1 Hz repetition rate and a pulse duration of 10 ms. d) Viability of electroporated cells pulsed without cargo and assessed 24 hours later. No significant differences in viability were observed at a given voltage (N=5). Error bars indicate ± standard deviation. *p < 0.05, ** p < 0.01, *** p <0.001.

We first examined the extent to which EP-mediated uptake of small fluorescent molecules in Jurkats is affected by pre-exposure to SμG in a rotating wall vessel bioreactor (RWV) (Fig. 1a,b). Uptake of exogenous propidium iodide (PI) decreased significantly (p < 0.05) by approximately 20% following 2 hours of exposure to SμG at 1400, 1500, and 1600V compared to both control groups (Fig. 1c). Identification of this range of interest is important because it corresponds to the field amplitudes that produced significant (> 25%) but not total (< 90%) PI uptake, leaving room for observation of differences induced by SμG. Significant differences in uptake between static and dynamic control (DC) groups were only observed at 1700V. The DC group was used to account for dynamic fluid motion within the rotating vessel by applying the same rotation rate as SμG, but with an axis of rotation parallel to gravity (i.e., on the horizontal plane). Although increasing voltage generally resulted in reduced cell viability, there were no significant differences between tests groups at a given voltage within and beyond the voltage range of interest (Fig 1d).

### Simulated Microgravity Exposure Reduces Transfection Efficiency

**Figure 2:**
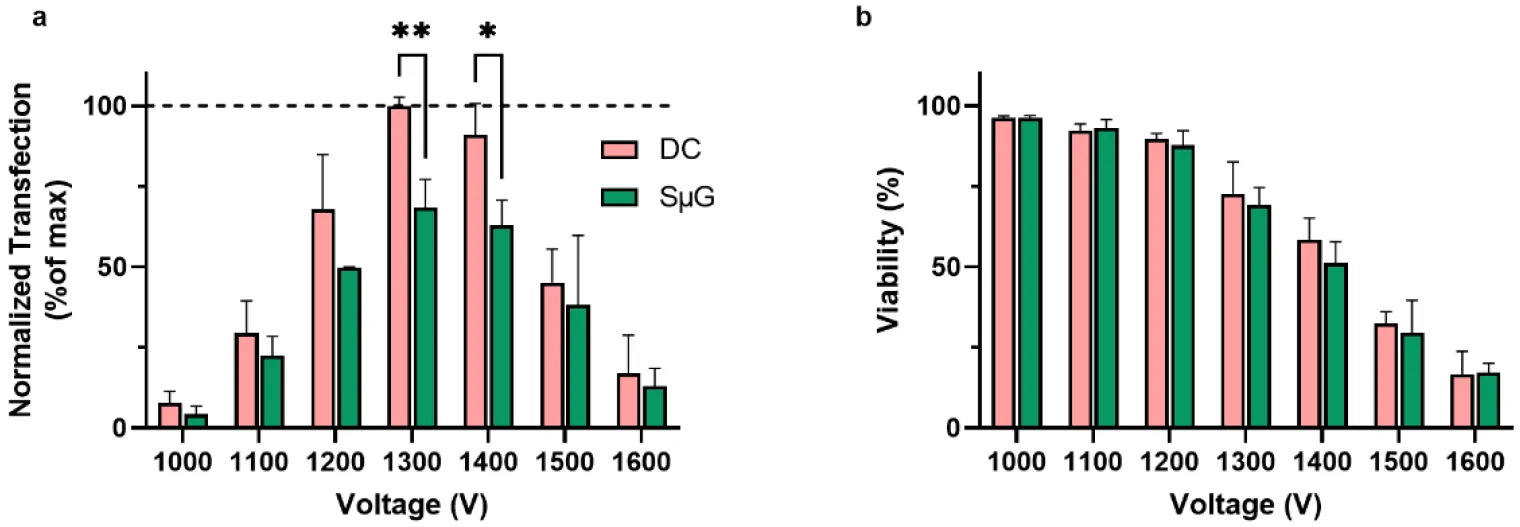
Jurkat cells exposed to SμG for two hours followed by transfection with an eGFP plasmid show reduced transfection efficiency but unchanged viability compared to the dynamic control. Cells were pulsed three times with 1 Hz frequency with a voltage ramp ranging from 1000 to 1600V and a pulse duration of 10 ms. a) Percentage of cells transfected normalized to the DC group at 1300V (24.05 ± 1.08% transfection efficiency) and b) their viability over increasing pulse amplitude. Images were taken 24 hours following transfection. Viability was not significantly impacted by microgravity for any voltage tested (p = 0.2468) but decreased with increasing voltage (p < 0.0001) . For both panels, N=3, error bars indicate ± standard deviation. *p < 0.05, **p < 0.01, ***p <0.001.

Next, we explored the effects of SμG exposure on transfection efficiency. To accomplish this goal, Jurkats were electroporated in the presence of plasmid DNA (pEGFP-N1) following exposure to SμG or DC for two hours. This experiment differs from our fluorescent molecule uptake as the cargo is larger, and successful transfection requires the transport of DNA to the nucleus. In the DC group, cells electroporated with 1300V showed the maximum transfection efficiency (24.05 ± 1.08%) across all experiments; other results were normalized to this value to facilitate comparison. We observed consistently lower transfection for cells exposed to SμG; notably, we found a 31% decrease in transfection efficiency at 1300V (p < 0.01) and 1400V (p < 0.05) for SμG exposure compared to the DC group (Fig 2a). Like our results above in Figure 1, we show no significant effects of microgravity on the viability of the cells at 24 hours following EP (p > 0.05; Fig 2b).

**Figure 3:**
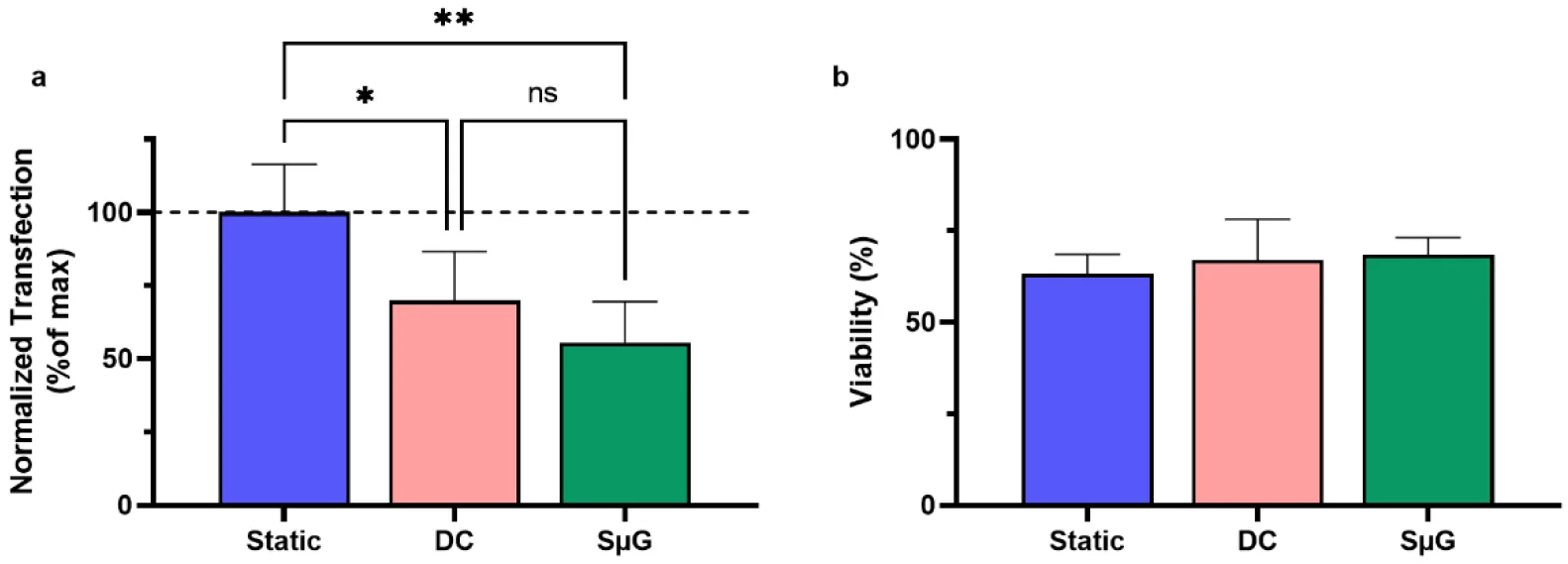
Jurkat cells exposed to SμG or dynamic control for 24 hours followed by transfection with an eGFP plasmid both experience reduced transfection compared to static conditions. Cells were pulsed three times with 1 Hz frequency with a field strength of 1300V and pulse duration of 10 ms. a) Percentage of cells transfected normalized to static conditions with a 1300 V pulse (8.54 ± 1.46% transfection efficiency) and b) their viability 24 hours following transfection. N=4, error bars indicate ± standard deviation. *p < 0.05, **p < 0.01.

To investigate the effect of a longer duration in microgravity, Jurkats were exposed to SμG for 24 hours prior to being transfected with plasmid DNA. Informed by our results in Figure 2, we tested a single amplitude (1300V), which previously led to the highest percentage of transfected cells. These results, as seen in Figure 3, show a 34% reduction in the percentage of cells successfully transfected in the DC group versus the static control, along with a larger decrease in the SμG group of 45% compared to the static control group (Fig 3a). No significant differences in viability were observed between groups 24 hours following EP (Fig 3b).

### Simulated Microgravity Exposure Induces Actin Remodeling in Jurkats

Thus far, we had observed a reduction in both uptake of exogenous molecules and transfection efficiency in Jurkats following 2 hours of exposure to SμG, but after 24 hours of exposure we observed a non-significant difference in transfection efficiency between DC and SμG groups, with both groups deviating from a static control. In our previous work, we observed that the reorganization of F-actin led to increased cell spreading which correlated with decreased uptake of fluorescent molecules in Jurkat cells exposed to SμG [18]. Disruption and forced remodeling of F-actin has been shown to influence the uptake of exogenous molecules [28]. Additionally, cell spreading is known to be modulated by changes in actin polymerization dynamics given that F-actin is bundled into lamellipodia structures that protrude from the cell membrane during cell spreading [33] and it has been shown that cell spreading is associated with F-actin dynamics and reorganization in T-cells [34]. To investigate actin remodeling dynamics and cell spreading following 2 or 24 hours of exposure to SμG, DC, and static conditions, we stained fixed Jurkats plated on glass bottom dishes with phalloidin. We then imaged and quantified cell size and shape to assess the effect of SμG exposure on actin reorganization.

**Figure 4:**
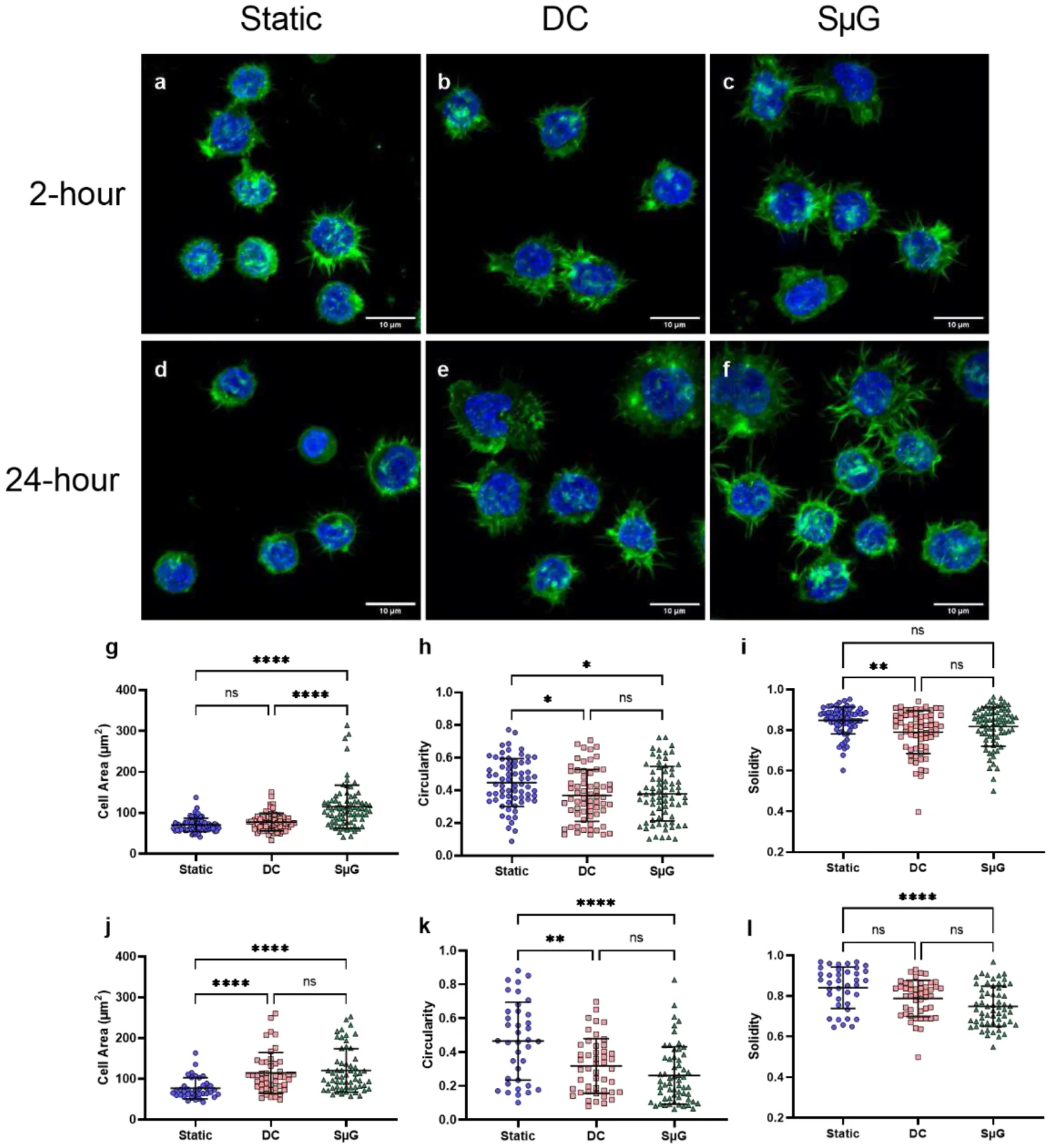
Z-stack maximum-intensity projection (MIP) images of phalloidin (green) and DAPI (blue) staining of F-actin in Jurkats exposed to SμG, DC, or static conditions for two hours (a-c) or 24 hours (d-f) show altered morphology. Quantification of cell area, circularity, and solidity as a surrogate metric for cytoskeletal reorganization after two hours (g-i) (N > 50 cells per condition) or 24 hours (j-l) (N > 40 cells per condition) of exposure to simulated microgravity. Scale bars indicate 10 μm. Error bars indicate ± standard deviation.* represents p < 0.05, ** p<0.01, *** p <0.001, *** p < 0.0001.

Phalloidin staining of Jurkats showed a 35% increase in average cell area following SμG exposure for two hours (Fig 4g) compared to both static and dynamic control groups (p < 0.0001). However, after 24 hours of exposure, cells in the DC and SμG groups were significantly larger than the static control, but not statistically different from each other (Fig 4j). Cell shape was quantified using circularity (Fig 4h,k); cells exposed to two hours of DC and SμG were significantly less circular in morphology compared to the static condition (p < 0.05). DC and SμG groups showed an even further reduction in circularity given 24 hours of exposure showing that longer exposure durations led to more irregularity in cell shape (Fig 4k). Solidity was also quantified to compare the ‘spikey’ shape of the cytoskeleton with a lower value indicating the presence of irregular protrusions (Fig 4i,l); two hours of exposure in the DC condition showed a significant decrease in solidity (p < 0.01) compared to the static group while SμG exposure did not show a significant decrease (p > 0.05). 24 hours of exposure in DC and SμG groups led to a decrease in solidity compared to the static group, however, the decrease in the SμG group was statistically significant (p < 0.0001) while the decrease in the DC group was not significantly different to either group (p > 0.05).

**Figure 5:**
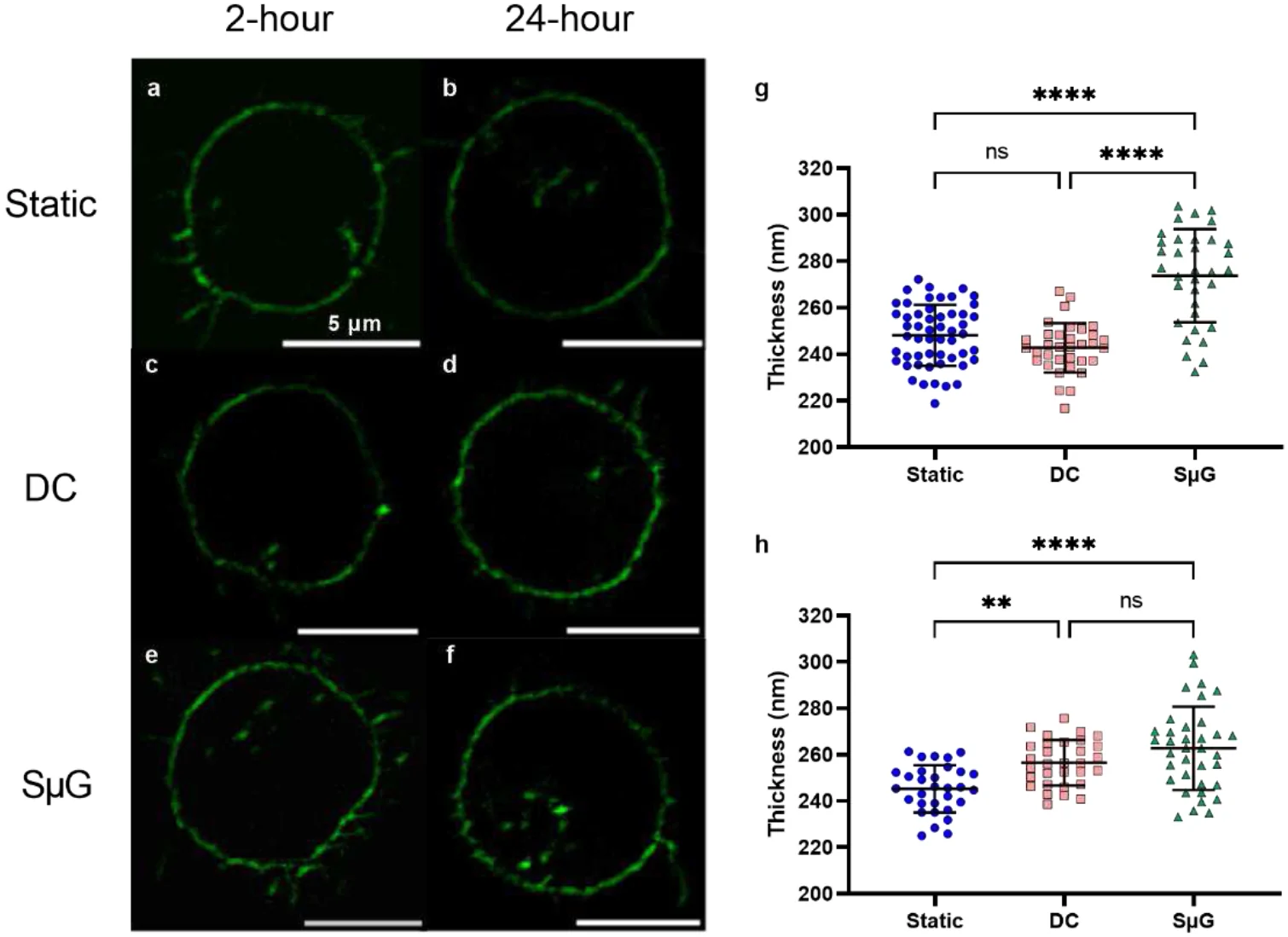
Z-stack slices of F-actin signal in Jurkats exposed to SμG, DC, or static conditions for two or 24 hours showing altered cortical actin thickness. a-f) Representative cortical actin of Jurkats after two-hour (a,c,e) or 24-hour (b,d,f) exposure to static, DC, or SμG conditions. Quantification of cortical actin thicknesses for g) two- or h) 24- hour exposure duration. Scale bars indicate 5 μm. N > 30 cells per condition. Error bars indicate ± standard deviation. ** represents p < 0.01, **** p < 0.0001.

We further investigated the role of F-actin reorganization in Jurkats by investigating changes in cortical actin thickness in response to SμG. Disruption (i.e., depolymerization) of cortical actin has been previously shown to increase transfection efficiency in other cell types [35]. Thus, because we observed decreased transfection efficiency following SμG exposure, we would expect to see increased cortical actin thickness if cortical actin plays a major role in regulating transfection after microgravity exposure. Fixed Jurkats were again stained with phalloidin, and images were collected using a laser scanning confocal microscope. Cortical thickness is reported as full width at half maximum (FWHM) thickness of the fluorescence signal from cortical actin, as determined using a line profile across the diameter of the cell in FIJI. Our results show that two hours of SμG exposure led to a 10% increase in the cortical actin thickness (Fig 5g), which was significantly different from DC and static controls (p < 0.0001). Additionally, after 24 hours of exposure (Fig 5h), the DC group had thickened cortical actin that was not significantly different compared to cells exposed to SμG for 24 hours (p > 0.05).

### F-actin Can be Targeted to Induce or Reverse the Effects of Simulated Microgravity

**Figure 6:**
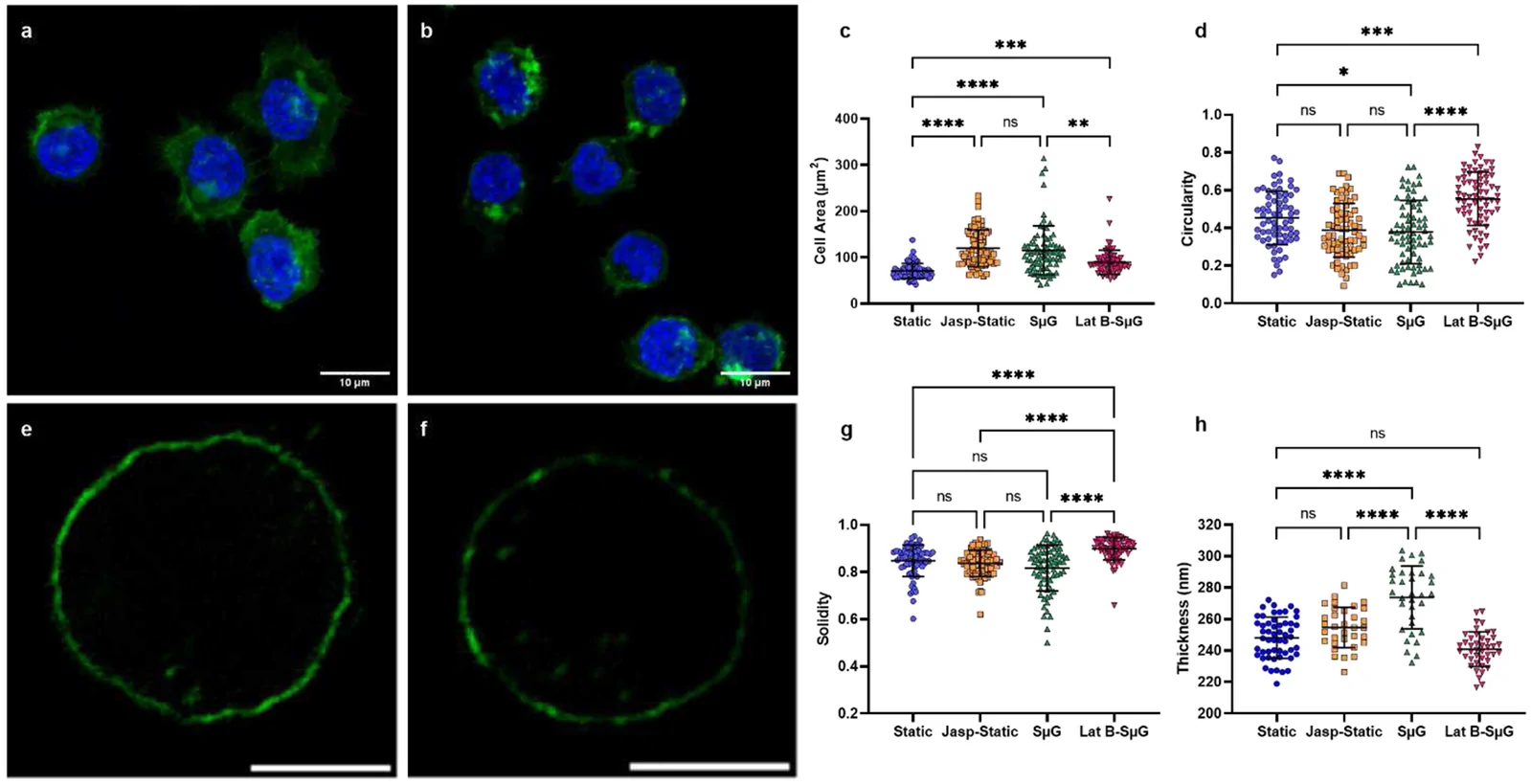
The Jurkat cytoskeleton can be modulated to mimic or prevent the effects of microgravity on F-actin organization. Images show Jurkat cells treated with F-actin targeting drugs for two hours in combination with the specified mechanical environment. Phalloidin staining of Jurkats a,e) treated with 50 nM jasplakinolide in static conditions and b,f) Jurkats treated with Lat B for two hours during SμG. a,b) Max intensity projection confocal fluorescence images showing cell area (scale bars indicate 10 μm) and e,f) a representative single slice of a confocal Z-stack showing cortical actin thickness (scale bar indicate 5 μm). Quantification of cell area (c), circularity (d), and solidity (g) (N > 50 cells per condition). h) Quantification of cortical actin thickness (N > 30 cells per condition). Error bars indicate ± standard deviation. *p < 0.05, ***p < 0.001, ****p < 0.0001.

Next, we investigated the correlation we observed between reduced transfection efficiency and increased cell spreading and cortical actin thickening. In these experiments, cells were treated with either an F-actin promoting (Jasplakinolide) or disrupting (Latrunculin B) drug prior to transfection to either simulate or counteract the effects of SμG under static conditions, respectively. F-actin disruption in Jurkats has been shown to decrease cell area [36] while promoting F-actin has been shown to increase cortical actin thickness in HeLa cells [37]. 50 nM Jasplakinolide was administered for two hours during static conditions to promote F-actin polymerization and mimic the effects of SμG that led to increased cell area and cortical actin thickness. 250 nM Latrunculin B (Lat B) was administered for two hours during SμG to disrupt F-actin polymerization and counteract the increase in cell area and cortical actin thickness. Our results show success in promoting cell area in a static culture treated with jasplakinolide and decreasing cell area and cortical actin thickness in SμG by treating with Lat B (Fig 6h). The treatments also caused significant changes to cell morphology (Fig 6d,g): compared to the typical static condition, jasplakinolide treated cells (Fig 6a) were more irregularly shaped reminiscent of cells exposed to SμG. On the other hand, cells treated with Lat B and exposed to SμG (Fig 6b) had smoother edges, higher circularity (Fig 6d), higher solidity (Fig 6g), and reduced cortical actin thickness (Fig 6h) than untreated SμG cells. However, jasplakinolide treatment did not significantly increase cortical actin thickness in static conditions (Fig 6h), which may be due to jasplakinolide and phalloidin molecules competing for F-actin binding sites leading to interference in the fluorescent signal [38]. We strongly suspect that this is the case, as jasplakinolide has been shown to increase cortical actin thickness when utilizing similar concentrations and exposure durations [37].

### F-actin Targeting can Modulate Transfection Outcomes While Preserving Viability

**Figure 7:**
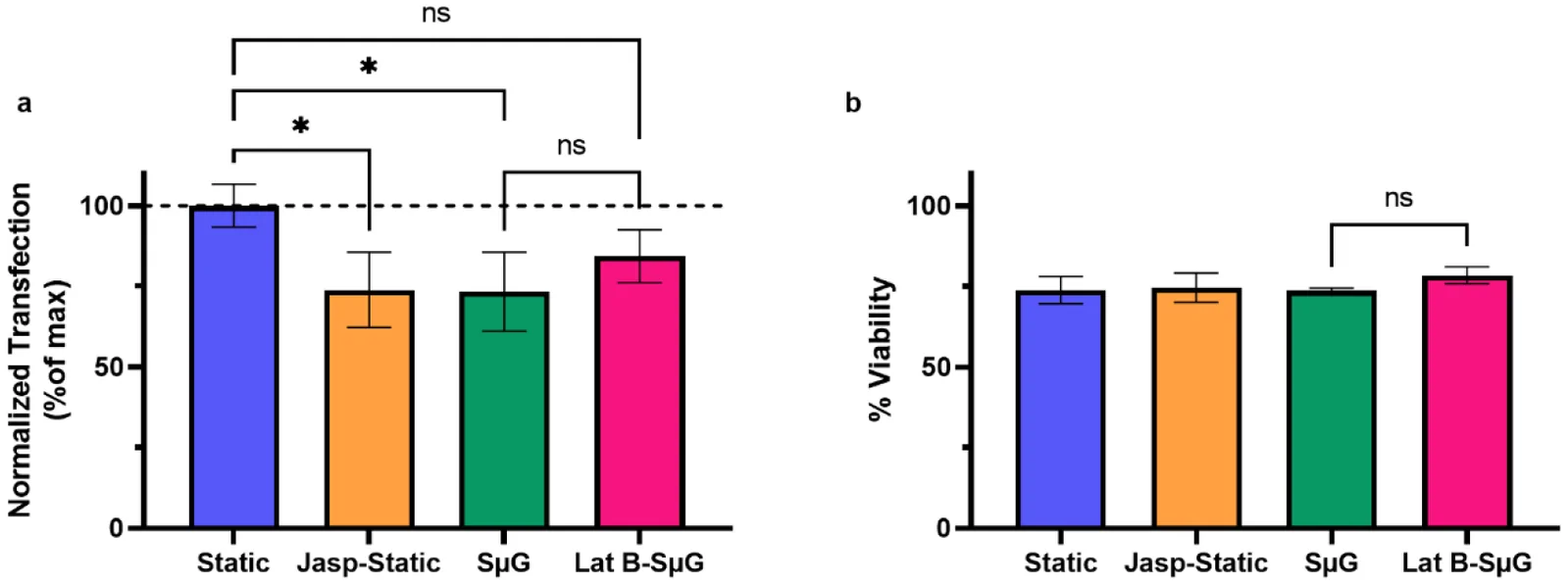
Jurkat cells treated with F-actin targeting drugs in the specified mechanical exposure condition for two hours followed by transfection with an eGFP plasmid. Cells were electroporated with a 1300V pulse at 1 Hz with 10 ms duration applied 3 times. a) Percentage of cells transfected normalized to static conditions (13.47 ± 1.12% transfection efficiency) and b) viability of transfected cells 24 hours following EP. N=3, error bars indicate standard deviation, *p < 0.05.

To determine whether the actin reorganization changes observed from drug treatment corresponded to functional EP outcomes, Jurkats were treated as described for the previous experiment (Fig 6), followed by transfection with eGFP plasmid as described for Figures 2 and 3. Our results show success in using jasplakinolide to induce a decrease in transfection efficiency similar to SμG: jasplakinolide treated cells show a 27% decrease in transfection efficiency compared to untreated cells in the static condition (Fig 7a). However, pretreatment with Lat B did not completely prevent the decreased transfection efficiency caused by exposure to SμG; a minor, nonsignificant increase of 7% was observed compared to the SμG group. Cell viability was not significantly affected by drug or mechanical treatments (Fig 7b).

## Discussion

In this investigation, we show that SμG exposure can impact both the physical uptake of exogenous molecules as well as plasmid DNA transfection efficiency facilitated by EP. Our experiments showed no difference in viability between standard and simulated microgravity conditions following EP, meaning that adaptations to SμG did not appear to affect cellular resilience after EP. We further investigated the effects of SμG on the reorganization of actin and observed an increase in cell area and thickening of cortical actin in microgravity groups at two hours of exposure and in both microgravity and dynamic control groups at 24 hours of exposure. These changes in cytoskeletal organization correlated with decreased uptake and transfection efficiency and suggest that cytoskeletal remodeling plays a role in transfection efficiency under SμG conditions. Furthermore, we showed that promoting polymerization of F-actin under static conditions allowed us to mimic the effects of SμG on actin organization and transfection efficiency. When we disrupted F-actin during SμG, the drug treatment showed a small but ultimately insignificant improvement in transfection efficiency compared to the SμG group.

Our transfection efficiency results are consistent with the transfection experiment performed aboard the ISS that showed decreased transfection efficiency in human iPSCs and human iPSC-derived fibroblasts using lipofectamine to deliver plasmid DNA [19]. Thus, it is likely that the altered mechanical environment of both true microgravity and SμG play a significant role in transfection efficiency. Exposing cells to altered mechanical environments has previously been shown to influence transfection efficiency. For example, cyclical stretching following EP led to an increase in transfection efficiency through the remodeling of the cytoskeleton [16]. A similar study using cyclical stretching showed that stretching frequency could be tuned to optimize transfection as the authors demonstrated that a stretching rate of 0.1 Hz led to higher transfection efficiency compared to 0.5 Hz [39]. These results indicate that adaptations to new mechanical environments can alter transfection efficiency but may require tuning to achieve desirable outcomes.

In our uptake and transfection experiments, we observed altered uptake and transfection efficiency in cells exposed to SμG at various voltages, while viability remained unaffected. The viability results differ from a previous study in yeast, which showed increased survivability after EP due to SμG exposure [17]. Cognizant of this study, we had initially hypothesized that improved survivability to electric pulses could allow for the delivery of more intense pulses under microgravity conditions, potentially leading to increased transfection efficiency without the downside of reduced viability. However, in this work, we observed that SμG induces a reduction in transfection efficiency without impacting viability in Jurkat cells. This contrast in viability outcomes following EP may be due to differences in cellular composition between mammalian and fungal cells as yeast cells lack a cortical actin structure [40]. Future work will explore whether these effects are cell type and species specific, to include whether the effects we have observed here are consistent between mammalian cells, bacteria, and fungi.

Our investigation into F-actin reorganization dynamics showed an increase in cortical actin thickness and cell area following SμG. In another study, Jurkats exposed to SμG for 24 hours showed increased B-actin expression, which may be implicated in the morphological changes we have observed in F-actin organization [32]. From these results, we attempted to offset this increase in actin polymerization by treating Jurkat T-lymphocytes with Lat B during SμG. We used a low concentration of Lat B so as to not overly disrupt polymerization of actin, which could also result in a further reduction of transfection efficiency [29]. This treatment led to a nonsignificant increase in transfection efficiency compared to the SμG group while also showing that the low concentration of F-actin targeting drugs was capable of modulating transfection efficiency while preserving viability given EP. These results indicate that further optimization of the concentration or duration of exposure may be required to fully offset the reduced transfection efficiency given SμG exposure. Additionally, our results show agreement in that promoting F-actin polymerization with jasplakinolide led to reduced transfection efficiency [29], similar to the effect of SμG exposure. It is believed that this reduction may be caused by denser actin networks disrupting intracellular plasmid trafficking [41]. However, our PI uptake results show that SμG exposure led to a decrease in uptake of about 20%, which does not require intracellular trafficking to the nucleus to make the cell fluoresce, while transfection efficiency was reduced by 31%. This indicates that the reduced transfection efficiency is likely a combination of effects where denser actin networks inhibit exogenous molecule uptake and trafficking to the nucleus. While our results clearly point toward the involvement of actin reorganization in microgravity as a potential driver for reduced transfection efficiency, more rigorous testing is needed to fully elucidate how cells adapt to altered mechanical environments and how these adaptations can potentially influence gene transfer techniques.

Because we simulated the effects of microgravity, this study has notable limitations. While using ground-based microgravity analogues have been shown to suitably model the effects of microgravity, phenomena may differ from those observed under true microgravity experienced during spaceflight [42, 43]. The goal of using an RWV is to minimize shear forces experienced by the cells during vessel rotation, but it does not completely negate these forces. Based on a computational study with similar vessel size, we estimate the shear forces in this work to be less than 10 mPa, whereas in true microgravity, shear forces approach zero [44, 45]. Because Jurkats are known to be sensitive to mechanical stimulation [31, 32] we incorporated a dynamic control group in our experiments to account for the effects of induced fluid shear forces in standard gravity. While we showed differences in transfection efficiency and F-actin organization between microgravity and DC groups given two hours of exposure, these differences were diminished by 24 hours of exposure, suggesting that minor shear forces may play a role in the biological effects induced by our microgravity analogues.

Another limitation of this study is the absence of cosmic radiation during SμG exposure. Exposure to cosmic radiation is expected to negatively impact cell health and plasmid integrity during extended durations in spaceflight, which may further complicate in-space transfection efficiency [46, 47]. Future studies could more closely mimic spaceflight conditions by utilizing radiation treated cells and plasmid DNA in addition to SμG. We expect that transfection efficiency may be further reduced by radiation exposure, though observing this phenomenon will require additional investigation.

In this study, Jurkat T-lymphocytes served as our model cell line due to their sensitivity to mechanical and electrical stimulation [31, 32]. However, adherent cell lines exhibit distinct adaptations to altered mechanical environments and thus EP outcomes following microgravity exposure may differ compared to suspension cells. Because both promotion and disruption of F-actin can decrease transfection efficiency [29], we anticipate that other cell types that undergo cytoskeletal remodeling in microgravity environments will exhibit altered transfection efficiency using EP. Future investigations to determine how the cytoskeletons of different cell types are altered by exposure to SμG would be valuable, especially since cytoskeletal reorganization dynamics can be cell-type specific [24, 48].

## Conclusion

In the future, gene transfer through techniques such as EP may represent an enabling technology that facilitates ISB to produce biomolecules for use on Earth or during long-term spaceflight. This investigation highlights how cellular adaptations to microgravity can affect transfection efficiency and provides further insights into strategies to offset these effects. Furthermore, our results show the need to improve our understanding of cellular adaptations to microgravity environments to learn how these adaptations may affect the practical use of established biotechnologies. Our investigation serves as a stepping-stone to future work on overcoming undesirable adaptations to microgravity that may hinder in-space biomanufacturing efforts.

## Methods

### Cell Culture and Simulated Microgravity Exposure

Jurkat, clone E6-1 (ATCC TIB-152) cells with passage number between 10-20 were used for EP experiments and were cultured in RPMI 1640 medium supplemented with 10% fetal bovine serum and 100 units/mL penicillinstreptomycin until cells reached a confluency of 1×10^6^ cells/mL and were passaged every two to three days. Cell counting was performed using a Countess II FL (Thermo-Fischer) according to the manufacturer’s instructions. Cells were cultured at 37° C in an incubator with an atmosphere containing 5% CO_2_. Simulated microgravity (SμG) experiments were performed in a rotating wall vessel (RWV) bioreactor (RCCS-4SC; Synthecon, Houston, TX) at 8 rotations per minute, in the upright position for SμG exposure. A dynamic control (DC) was performed while the RWV was on its side at the same rotation rate to produce mechanical stimulation and low shear forces under standard gravity conditions [49]. A static control group was also developed by placing cells in a dish in static conditions in standard gravity.

### Electroporation and Transfection

To electroporate cells, a Neon transfection system (Thermo-Fisher) was employed using custom EP buffers [50]. For exogenous molecule uptake, cells were resuspended in 1X PBS to achieve cell density of 5×10^6^ cells/mL and were exposed to SμG, DC, and static conditions for two hours. Cells were then collected and mixed with propidium iodide (PI) to a final concentration of 75 μM prior to pulsing [51]. Cells were pulsed in the presence of PI to facilitate the uptake of this otherwise exclusionary dye. Pulsed cells were then dispensed into complete media. 5 μM Hoechst 33342 was added to stain for nuclei, followed by a 30-minute incubation at 37° C with 5% CO_2_ prior to imaging. Cells were pulsed between 1000-1800V with 100V intervals using a fixed pulse length of 10 ms with three pulses applied at 1 Hz frequency. Because PI stains both dead and electroporated cells [52], we could not differentiate between cells that were dead and cells that were successfully electroporated. Therefore, we ran a separate experiment to evaluate cell viability by pulsing cells without PI. 24 hours after EP, 5 μM PI and 5 μM Hoechst 33342 were added to the cells to stain for dead cells and nuclei, respectively.

To investigate changes in transfection efficiency following microgravity exposure, cells were electroporated in the presence of plasmid DNA (pEGFP-N1). Cells were collected and resuspended in fresh complete media to achieve a cell density of 1×10^6^ cells/mL. Cells were then incubated for 2 or 24 hours while exposed to SμG, DC, or static conditions. Following exposure, cells were centrifuged at 200 rcf for 4 minutes, washed with PBS and centrifuged again, then resuspended in BTXpress Cytoporation^®^ Medium T4 (BTX; Cat. No. 47-0003) to achieve a cell suspension of 2×10^7^ cells/mL and were then electroporated in the presence of 2 μg of plasmid DNA per 10 μL of cell suspension according to the manufacturer’s instructions. Cells were then transferred to antibiotic free complete media for 24 to assess transfection efficiency. 5 μM PI and 5 μM Hoechst 33342 were included the day of imaging to stain for dead cells and nuclei, respectively.

Live cell fluorescent imaging was performed at 20X magnification using an EVOS FL microscope for imaging of the stained or transfected cells using RFP, GFP, and DAPI light cubes. Images were then analyzed using FIJI [53] to determine the percentage of RFP+ (PI uptake or dead) cells compared to the number of cells (DAPI+). Transfection efficiency was determined similarly by finding the percentage of GFP+ cells relative to the total number of DAPI+ cells. Viability was assessed by finding the percentage of RFP+ relative to the total number of DAPI+ cells.

### Plasmid DNA

The pEGFP-N1 plasmid was originally obtained from Clontech (now Takara Bio; Cat. No. 6085-1). The plasmid was electroporated into electrocompetent XL1-Blue E. coli cells and amplified under kanamycin selection (50 μg/mL). Endotoxin-free plasmid DNA was purified using the QIAGEN EndoFree Plasmid Maxi Kit (Cat. No. 12362) according to the manufacturer’s instructions and resuspended in ultrapure water.

### Cytoskeletal Staining

F-actin organization was analyzed using a phalloidin dye (Thermo-Fisher, excitation: 495 nm; emission: 518 nm) to determine changes in cytoskeletal organization and cortical actin thickness. Following exposure to SμG, DC, or static conditions in complete media, Jurkats were centrifuged and suspended in PBS. Jurkats were then plated on glass bottom 35 mm MatTek^™^ dishes and allowed to adhere to the surface for 20 minutes. PBS was aspirated and cell were fixed in 4% paraformaldehyde for 20 minutes and permeabilized with 0.2% Triton X-100 for 10 minutes. Cells were washed twice with PBS after each step. F-actin was then stained with phalloidin according to the manufacturer’s instructions followed by a 30-minute incubation. Fluoromount-G mounting media (Electron Microscopy Sciences) containing DAPI stain was then added and covered with a glass coverslip to preserve fluorescent signal. Z-stack images were collected using a Nikon TI-E2 A1 confocal microscope with a magnification of 60X using 405 and 488 nm lasers with a step height of 0.25 μm. Cell area, circularity, and solidity were quantified using the ‘Analyze Particles’ function in FIJI.


(1) $$Circularity=\frac{4\pi A}{p^{2}}$$

(2) $$Solidity=\frac{A_{c}}{A_{h}}$$

Circularity is defined as the ratio of the area of the object (*A*) to the area of a circle with the same perimeter (*p*) and solidity is defined as the ratio of the area of the object (*A_c_*) to the area of the convex hull (*A_h_*) [54].Cortical actin thickness was quantified using FIJI via the full width at half maximum (FWHM) of the signal to approximate the thickness, as determined from a line trace across the diameter of a cell. At least seven measurements were collected and averaged for each cell.

### F-actin Targeting Drug Treatment

F-actin targeting drugs jasplakinolide and latrunculin B were administered to promote or disrupt F-actin polymerization, respectively and were initially dissolved in DMSO. Cells cultured in static conditions were treated with 50 nM jasplakinolide for two hours in complete culture media. Cells in the RWV were treated with 250 nM latrunculin B for 2 hours during SμG exposure. The final concentration of DMSO in treated cultures was maintained below 0.01% (v/v). Following exposure, cells were centrifuged and washed with PBS prior to further processing for fixation or transfection.

### Statistical Analysis

Experimental testing included at least 3 replicates per condition and error bars indicate standard deviation. One-way ANOVA or two-way ANOVA with Tukey post-hoc testing for multiple comparisons were used at a confidence interval of 95%. Statistical testing was performed using GraphPad Prism 9.4.1.

## Data Availability

The data that support the findings of this study are available from the corresponding author upon reasonable request.
